# Constrictive Pericarditis: An Unusual Presentation of Rheumatoid Vasculitis

**DOI:** 10.7759/cureus.21643

**Published:** 2022-01-26

**Authors:** Adnan Kharsa, Medhat Chowdhury, Bryan E-Xin Tan, Mohammad Abu Sheikha, Bipul Baibhav

**Affiliations:** 1 Internal Medicine, Rochester Regional Health, Rochester, USA; 2 Cardiology, Rochester Regional Health, Rochester, USA; 3 Cardiology, Sands-Constellation Heart Institute, Rochester Regional Health, Rochester, USA

**Keywords:** diffuse lymphadenopathy, constrictive pericarditis, rheumatoid vasculitis, leukocytoclastic vasculitis, pericardiectomy

## Abstract

We describe a case of rheumatoid vasculitis with an atypical presentation of constrictive pericarditis. A 51-year-old man who was previously admitted for diffuse lymphadenopathy, presented with chest pain and a lower extremity rash. Extensive workup including multimodality imaging, serology tests, and biopsy, resulted in the diagnosis of rheumatoid vasculitis.

## Introduction

Constrictive pericarditis (CP) is scarring of the pericardium that can occur due to various causes that include idiopathic CP, viral illness, previous cardiac surgery, or less commonly connective tissue disorders [1,2]. Rheumatoid vasculitis (RV) is a serious extra-articular manifestation of rheumatoid arthritis (RA) in patients with a long-standing history of RA [3]. Involvement of cardiac tissue is present in 30% of patients with RV and this can manifest as pericarditis, myocarditis, coronary arteritis, or aortitis [3]. This article reports a patient who was initially found to have CP and later diagnosed with RV.

## Case presentation

A 51-year-old male presented to the emergency department with chest pain of four days duration. The pain was described as constant, non-radiating, and dull. The patient also reported a non-pruritic skin rash with similar onset, initially over the thighs, progressively extending to the lower abdomen. A review of systems disclosed progressive swelling of hands and legs. Physical examination was remarkable for jugular venous distension, bibasilar rales, and 2+ pitting edema of bilateral legs. No added sounds or friction rubs were noted. The abdomen was tender over the left lower quadrant with voluntary guarding. The patient had significant swelling and tenderness of bilateral hands. Violaceous rash extending from the thighs to the lower abdomen; slightly raised, non-blanching, and non-scaly.

A week before his presentation, he was admitted with non-radiating left-lower quadrant abdominal pain. Imaging including computed tomography (CT) scan of the chest and abdomen/ pelvis revealed diffuse lymphadenopathy involving the mediastinum, axillae, lower abdomen and pelvis, and a moderate-sized pericardial effusion. Echocardiography was remarkable for a dilated right atrium, dilated right ventricle with preserved systolic function, and a small circumferential pericardial effusion with mitral inflow respiratory variation without any evidence of tamponade physiology. Lymph node biopsies obtained from the right axilla and external iliac node subsequently were inconclusive.

Past medical history

Pertinent history was notable for bilateral carpal tunnel syndrome. He was recently evaluated for upper and lower extremities weakness and was diagnosed with sensorimotor polyneuropathy of indeterminate etiology. Pertinent family history disclosed a niece with juvenile arthritis. Social history disclosed a 15-year-pack history of smoking.

Differential diagnosis

The differential diagnosis of skin rash, diffuse lymphadenopathy, and pericardial effusion is broad and includes lymphoma or malignancy, rheumatological disorder, vasculitis, cutaneous amyloidosis, cutaneous sarcoidosis, or infectious process.

Further investigations

**Figure 1:**
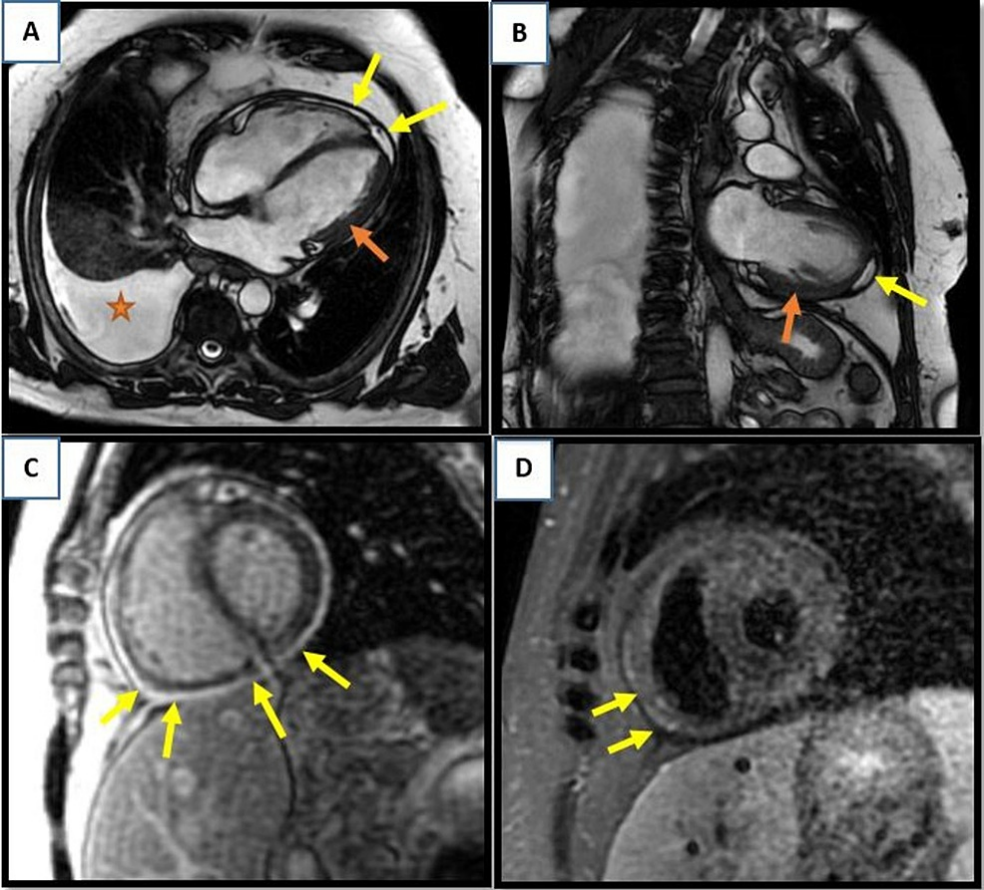
Cardiac MRI. (A) Cross-sectional four-chamber and (B) coronal steady-state free precession (SSFP) images showing thickened pericardium (red arrows), small pericardial effusion (yellow arrows), and right-sided pleural effusion (red star). (C) Diffuse pericardial delayed enhancement is consistent with pericardial inflammation (arrows). (D) T2-weighted image showing thickened pericardium (arrows).

**Figure 2:**
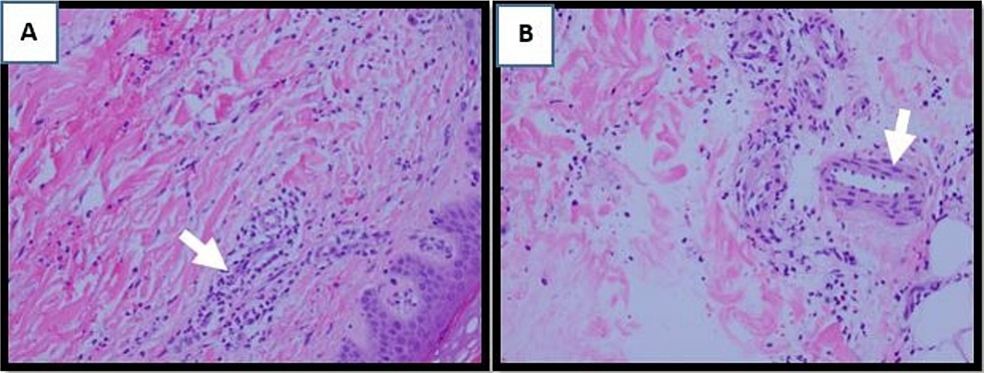
Skin biopsy results. The skin biopsy reveals (A) neutrophilic infiltration (arrow) of the dermis and extravasated red blood cells. It also shows (B) fibrinoid necrosis of the vessels wall (arrow). Findings are consistent with leukocytoclastic vasculitis.

Laboratory investigations were remarkable for mild normocytic anemia, elevated rheumatoid factor, and elevated anti-cyclic citrullinated peptide antibodies (Table 1). Electrocardiography (ECG) revealed nonspecific T-wave changes with negative biomarkers. A coronary angiogram revealed normal coronary arteries. Cardiac MRI revealed thickened pericardium circumferentially with evidence of pericardial tethering, ventricular interdependence, and diffuse pericardial hyperenhancement suggesting pericardial inflammation (Figures 1A-1D). A punch biopsy of the skin rash revealed dermal neutrophils, extravasation of red blood cells, and focal fibrinoid vascular change (Figures 2A, 2B). Findings were consistent with leukocytoclastic vasculitis.

**Table 1:** Laboratory investigations. Hb: hemoglobin. ESR: sedimentation rate. CRP: c-reactive protein. RF: rheumatoid factor. Anti-CCP: anti-cyclic citrullinated peptide antibodies.

Blood test	Patient’s lab value	Reference range
Hb	10 g/dL	13.0-18.0 g/dL
ESR	74 mm/hr	0.0-23.0 mm/hr
CRP	109 mg/L	0.0-10.0 mg/L
RF	1700 IU/mL	0.1-13.9 IU/mL
Anti-CCP antibodies	> 300 U/mL	< 3 U/mL

Management

The patient was diagnosed with CP secondary to RV and was started on pulse methylprednisolone treatment. He was then transitioned to oral prednisone on discharge.

Follow-up

The patient remained symptomatic for several months despite medical management, including the addition of methotrexate and later sulfasalazine to his regimen. A repeated cardiac MRI revealed persistent pericardial inflammation. He was eventually referred to cardiothoracic surgery and underwent pericardiectomy. He tolerated the surgery well with an improvement in his clinical status.

## Discussion

CP is a pericardial disease that occurs due to scarring and loss of elasticity of the pericardium. Inelastic pericardium does not expand and thus prevents the physiological decrease in intrathoracic pressure during inspiration to be transmitted to the chambers of the heart. This leads to elevated filling pressures of the right ventricle, which in turn shifts the interventricular septum towards the left ventricle (LV) impairing LV filling [1].

CP can occur after any pericardial process. The majority of cases are attributed to idiopathic CP, viral illness, and previous cardiac surgery [2]. Other common causes include radiation therapy to the chest and post-infectious (tuberculous) pericarditis. Less common causes also include trauma, uremia, drugs, and malignancies. Rarely, CP may be a manifestation of vasculitis and connective tissue disorders.

RV is one of the most severe extra-articular complications of RA and is almost always seen in patients with long-standing and advanced RA [3]. In our case, the diagnosis of RV was made in the absence of long-standing RA. Skin is most frequently affected, with a prevalence of 90% among all patients with RV. This is followed by peripheral nervous system involvement, with various manifestations including sensory, motor, and mixed polyneuropathy. The involvement of cardiac tissue is the third most prevalent and is present in about 30% of patients with RV [3]. Pericarditis is a common manifestation, but myocarditis, coronary arteritis, and aortitis can also occur.

History and physical examination are essential in the diagnosis of CP. EKG may show non-specific ST-T changes. Laboratory investigations may suggest an inflammatory process. Echocardiography is usually the initial imaging test. It assesses pericardial thickness, the motion of the pericardium and the myocardium, motion of the septal wall, ventricular interdependence, and cardiac chambers filling pressures. A combination of findings on echocardiograms can have up to 89% sensitivity and 95% specificity for the diagnosis of CP [4]. Cardiac MRI provides morphological assessment and hemodynamic characteristics in patients with CP. It also determines the extent of the pericardial inflammation and provides input to guide treatment [5]. CT scan can reveal the presence of pericardial calcification in 25% of patients, and aid in the pre-operative assessment [6].

Histopathological demonstration of vasculitis is the confirmatory diagnostic test. Findings of infiltration of the vessel wall by inflammatory cells and neutrophilic nuclear remnants along with accompanying fibrinoid necrosis of the vessel wall are present in most patients with RV [7].

Treatment options for CP include medical and surgical therapy. Medical therapy can be considered for specific etiologies like tuberculous pericarditis and in cases with transient pericarditis. Medical therapy is also used to control symptoms of heart failure (mainly diuretics) when surgery is not feasible. Otherwise, pericardiectomy is the mainstay treatment for early refractory CP and chronic CP. It has high perioperative morbidity and mortality, and thus it should be considered cautiously. 83% of patients who undergo pericardiectomy are free of clinical symptoms [8], with an overall survival rate of around 60% at 10 years [9].

Due to the paucity of randomized trials and rarity of occurrence of RV, there are no established guidelines regarding treatment. Patients usually receive high doses of corticosteroids and immunosuppressive agents. A large, single-center retrospective study reported that two-thirds of patients were treated with corticosteroids and one-third with cyclophosphamide. Other immunosuppressive agents were less commonly used. Biologic agents, including anti-TNF agents, rituximab, and anakinra were also used. Despite treatment, however, mortality rates remain high [10].

## Conclusions

CP occurs due to scarring and loss of elasticity of the pericardium. It rarely occurs as a manifestation of vasculitis. RV is an uncommon complication of RA that usually occurs in males with a long-standing history of RA. Despite treatment, both CP and RV pose treatment challenges and have poor survival outcomes.
